# Electron-Deficient Alkynes as Powerful Tools against Root-Knot Nematode *Melodogyne incognita*: Nematicidal Activity and Investigation on the Mode of Action

**DOI:** 10.1021/acs.jafc.0c00835

**Published:** 2020-09-14

**Authors:** Graziella Tocco, Kodjo Eloh, Antonio Laus, Nicola Sasanelli, Pierluigi Caboni

**Affiliations:** † Department of Life and Environmental Sciences, University of Cagliari, Cittadella Universitaria di Monserrato, Via Ospedale 72, 09042 Monserrato, Cagliari, Italy; ‡ University of Kara, Post Office Box 404, Kara, Togo; § Istituto per la Protezione delle Piante, Consiglio Nazionale delle Ricerche, Via G. Amendola 122/D, 70126 Bari, Italia

**Keywords:** electron-deficient alkynes, 3-butyn-2-one, dimethyl acetylenedicarboxylate, methyl propiolate, vacuolar-type H^+^-ATPase, *Meloidogyne incognita*, *Meloidogyne javanica*

## Abstract

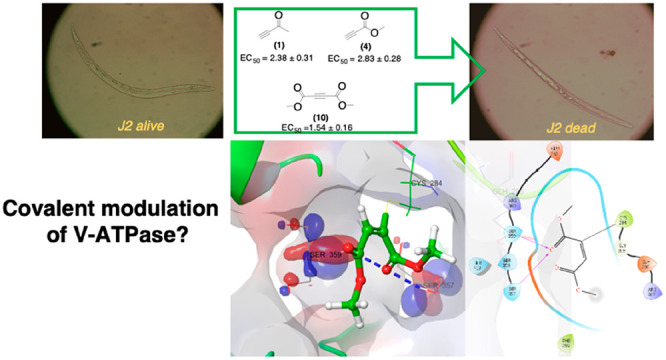

The present study reports on the powerful nematicidal activity of a series of electron-deficient alkynes against the root-knot nematode *Meloidogyne incognita* (Kofoid and White) Chitwood. Interestingly, we found that the conjugation of electron-withdrawing carbonyl groups to an alkyne triple bond was extremely proficient in inducing nematode paralysis and death. In particular, dimethylacetylenedicarboxylate (**10**), 3-butyn-2-one (**1**), and methyl propiolate (**4**), with EC_50/48 h_ of 1.54 ± 0.16, 2.38 ± 0.31, and 2.83 ± 0.28 mg/L, respectively, were shown to be the best tested compounds. Earlier studies reported on the ability of alkynoic esters and alkynones to induce a chemoselective cysteine modification of unprotected peptides. Thus, also following our previous findings on the impairment of vacuolar-type proton translocating ATPase functionality by activated carbonyl derivatives, we speculate that the formation of a vinyl sulfide linkage might be responsible for the nematicidal activity of the presented electron-deficient alkynes.

## Introduction

Phytoparasitic nematodes are considered highly damaging crop pests capable of attacking different plant organs and, therefore, hampering the life cycle of a great range of hosts. Root-knot nematodes belonging to the genus *Meloidogyne*, have the worst record because they are the world’s most damaging soil-borne pathogens. Specifically, *Meloidogyne incognita* and *Meloidogyne javanica* are the most detrimental crop parasites because they are able to infest the roots of almost all cultivated plants.^1^ It is estimated that nematodes are responsible of annual yield losses of roughly $157 billion USD worldwide.^2^

Throughout the past few decades, different strategies, such as crop rotation, biological control, soil solarization, and chemical nematicides, have been adopted in the attempt to reduce the spread of nematode infection.

At the present time, the development of new nematotoxicants still represents a challenge as a result of the presence of multiple environmental, field persistent, and toxicity issues. In fact, commercially available fumigants, despite their high volatility and consequent excellent field diffusing capacity, envisage repeated treatments as a result of their rapid dissipation and chemical decomposition.^3^ On the other hand, non-volatile nematicides, such as organophosphorus, notwithstanding their relatively simple soil application, are still extremely toxic for the environment and the operators.^4^ Moreover, problems related to the biology of the parasite, such as the low permeability of the cuticle of the nematode, justify the increasing demand of new chemicals with a high target specificity and selective mode of action.^5^ In this respect, V-ATPase seems to be a perfect biological target, because it is involved in the cuticle synthesis, nutrition, osmoregulation, and reproduction of the nematode.^6,7^

Our recent studies revealed significant alterations of the external cuticle of the root-knot nematode *M. incognita* after treatment with aromatic aldehydes, such as 2-naphthaldehyde and cinnamic aldehyde.^8^ It has been postulated that these damages were a consequence of a thioacetalization reaction between the nematicide carbonyl group and some thiol cysteine residues of the nematode protein exoskeleton. Moreover, it was also hypothesized that redox-active aromatic aldehydes, such as salicylaldehyde, able to generate reactive oxygen species (ROS), might have a role in hindering the functionality of V-ATPase and, therefore, affecting the osmoregulation of nematodes.^8,9^ More recently, as a support to this mechanism of action, we reported on the nematicidal activity of tulipaline A, 5,6-dihydro-2*H*-pyran-2-one,^10^ and a series of ketones,^11^ assuming a strict connection between their biological activity and their ability to hamper V-ATPase through the formation of covalent vinyl sulfide linkages.

This evidence prompted us to investigate the nematicidal activity of some selected acetylene derivatives, because they are known to be synthetic equivalents of aldehydes or ketones.^12^ In fact, alkynes can be rapidly converted into carbonyl compounds via hydration, because the −C≡C– group retains the same oxidation state of the −CH_2_CO– unit. On the other hand, alkynes suffer from a lack of polarization. Thus, the triple bond needs to be preactivated with electron-withdrawing substituents to interact with nucleophiles. In this respect, the conjugation of an alkyne functional group with ketone or ester functionalities seems to be the most amenable choice. Accordingly, in the present work, we speculated on the correlation between the chemical reactivity and the nematicidal activity of a series of selected alkynes, proposing a possible mechanism of action also with the support of docking analysis.

## Materials and Methods

### Chemicals

**Figure 1:**
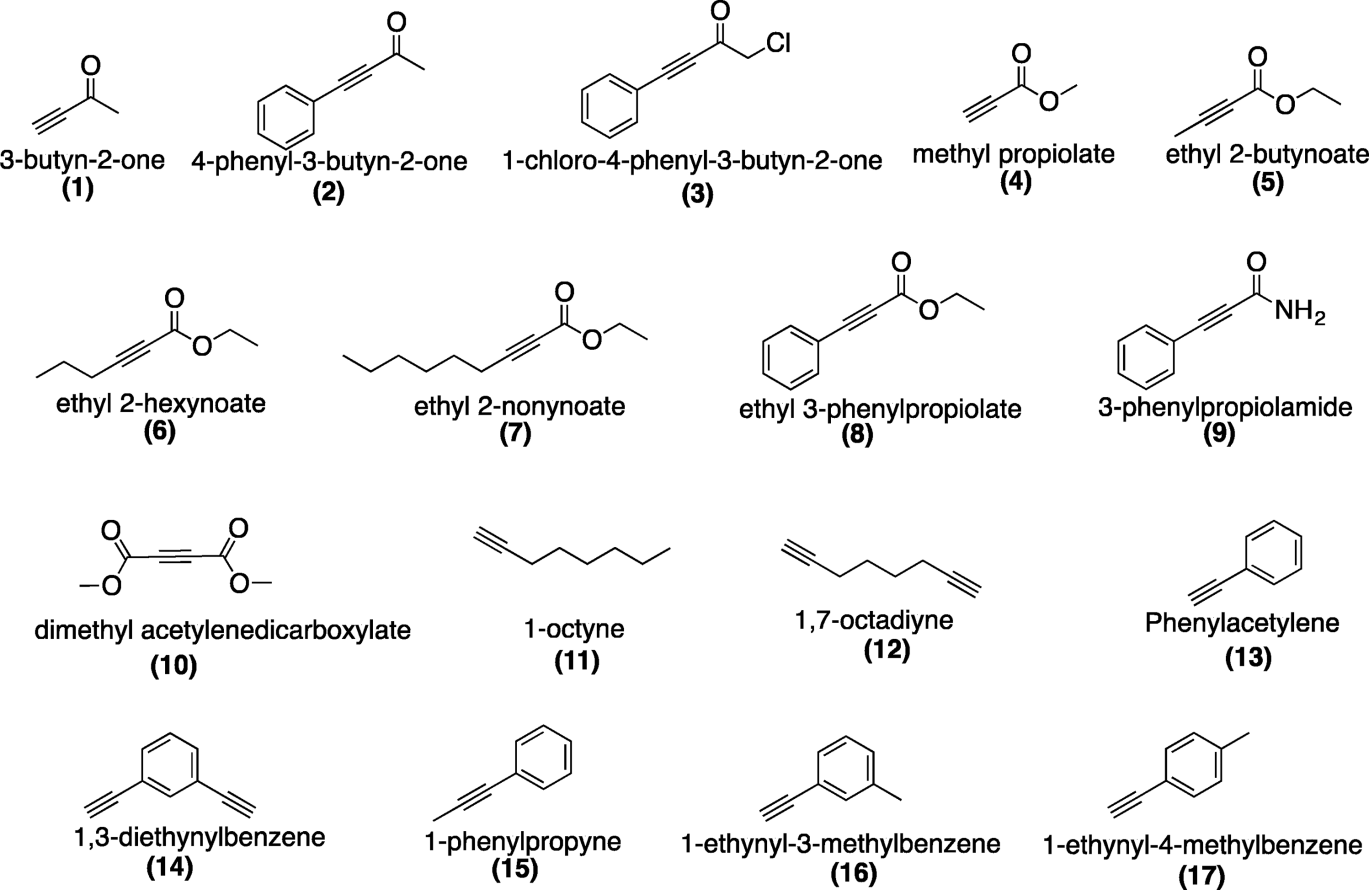
Chemical structures of nematicidal compounds used for bioassays against *M. incognita*.

The acetylene derivatives **1**, **2**, **4**–**8**, and **10**–**17** shown in Figure 1 were purchased from Sigma-Aldrich, Merck Group (Milan, Italy), Alfa Aesar, and Thermo Fisher Scientific. According to the literature, compounds **3**,^13^**9**,^14^**20**,^15^ and **21**(16) were prepared as follows.

### Experimental Chemistry

Reaction progress was monitored by thin-layer chromatography (TLC) using Aldrich silica gel 60 F254 (0.25 mm) plates. ^1^H and ^13^C nuclear magnetic resonance (NMR) spectra were recorded on a Varian Unity Inova 500 MHz spectrometer. High-resolution mass spectrometry (HRMS) spectra were recorded using an Agilent 6520 liquid chromatography quadrupole time-of-flight mass spectrometry (LC–QTOF–MS) system.

### Synthesis of 1-Chloro-4-phenyl-3-butyn-2-one (**3**)

A stirred solution of phenylacetylene (**13**, 14.7 mmol) in dry tetrahydrofuran (THF, 7.5 mL) was cooled at 0 °C. After 15 min, *n*-BuLi (2.5 M in hexane, 5.9 mL) was added dropwise under nitrogen, and the mixture was stirred for 30 min. To the so generated lithium acetylide, a solution of 2-chloro-*N*-methoxy-*N*-methylacetamide (9.8 mmol) in dry THF (5 mL) was added dropwise, and the reaction mixture was stirred for another 30 min, maintaining the temperature at 0 °C. Then, the reaction mixture was quenched with aqueous 1 N HCl. The layers were separated, and the organic layer was washed with water and brine and dried over Na_2_SO_4_. The solvent was removed under reduced pressure, and the crude material was purified by flash chromatography on silica gel (9:1 hexane/AcOEt), to give the pure product as orange sticky oil. Yield (%) = 75. ^1^H NMR (500 MHz, CDCl_3_): δ 7.64 (d, *J*_3_ = 7.5 Hz, 2H, ArH), 7.52 (t, *J*_3_ = 7.5 Hz, 1H, ArH), 7.44 (d, *J*_3_ = 7.5 Hz, 2H, ArH), 4.33 (s, 2H, CH_2_Cl). HRMS: calculated for C_10_H_7_ClO, 178.02; observed, 178.02.

### Synthesis of 3-Phenylpropiolamide (**9**)

Ethyl 3-phenylpropiolate (**8**, 5.74 mmol) was dissolved in 1.3 mL of 25% NH_3_/H_2_O (23 mmol) and stirred at room temperature for 24 h. Then, solvent and other volatile compounds were removed under reduced pressure at room temperature, obtaining the pure product as white solid [melting point (mp) = 107–109 °C]. Thus, a further purification was not necessary. Yield (%) = 90. ^1^H NMR [500 MHz, dimethyl sulfoxide (DMSO)]: δ 8.05 bs, 2H, CONH_2_), 7.58 (d, *J*_3_ = 7.0 Hz, 2H, ArH), 7.49–7.46 (m, 3H, ArH). HRMS: calculated for C_9_H_7_NO, 145.05; observed, 145.06.

### Synthesis of *N*-Benzyl 2-acetylamino-3-mercaptopropionamide (**20**)

To *N*-acetylcysteine (**18**, 6.14 mmol) in dichloromethane (DCM, 50 mL) and *N*,*N*-dimethylformamide (DMF, 6 mL) at 0 °C, *N*-hydroxybenzotriazole (HOBt, 6.76 mmol), benzylamine (**19**, 9.20 mmol), and *N*-(3-dimethylaminopropyl)-*N*′-ethylcarbodiimide (EDC, 6.76 mmol) were sequentially added. The reaction was brought to room temperature and stirred for 16 h. The solvent was removed *in vacuo*, and the pale-yellow crude product was recrystallized from CH_3_CN and H_2_O, to give the pure product (**20**) as a white solid (mp = 163–165 °C). Yield (%) = 58. ^1^H NMR (500 MHz, DMSO): δ 8.56 (t, *J*_3_ = 6.0 Hz, 1H, NHCH_2_), 8.22 (d, *J*_3_ = 8.5 Hz, 1H, NHCH), 7.49–7.32 (m, 2H, ArH), 7.26–7.23 (m, 3H, ArH), 4.59 (m, 1H, CHNH), 4.29 (t, *J*_3_ = 6.0 Hz, 2H, NHCH_2_), 3.13 (dd, *J* = 4.5 and 13 Hz, 1H, HCHSH), 2.90 (dd, *J* = 4.5 and 13 Hz, 1H, HCHSH), 1.89 (s, 3H, CH_3_CO). ^13^C NMR (500 MHz, DMSO): δ 173.14, 172.63, 142.30, 131.36, 130.21, 129.85, 55.25, 45.35, 25.73. HRMS: calculated for C_12_H_16_N_2_O_2_S, 252.09; observed [M + Na]^+^, 275.10.

### Synthesis of 2-Acetamido-*N*-benzyl-3-[(3-oxobut-1-en-1-yl)thio]propenamide (**21**)

A solution of mercaptopropionamide (**20**, 2 mmol) and 3-butyn-2-one (**1**, 2.5 mmol) in H_2_O/CH_3_CN (200 mL, 9:1) was prepared and stirred at room temperature for 16 h. Then, the mixture was extracted with DCM (3 × 40 mL), and the combined organic layers, previously dried over MgSO_4_, were filtered and concentrated under reduced pressure, to give a crude product as a pale yellow solid (mp = 131–133 °C) that was purified by flash chromatography on silica gel (9:1 hexane/AcOEt). Yield (%) = 82. ^1^H NMR (500 MHz, DMSO): δ 8.64 (t, *J*_3_ = 6.2 Hz, 1H, NHCH_2_), 8.20 (d, *J*_3_ = 8.5 Hz, 1H, NHCH), 7.51 (d, *J*_3_ = 9.8 Hz, 1H, SCH=CHCO), 7.37–7.29 (t, *J*_3_ = 7.4 Hz, 2H), 7.26–7.21 (m, 3H), 5.94 (d, *J*_3_ = 9.8 Hz, 1H, SCH=CH), 4.49 (m, 1H, CHNH), 4.26 (t, *J*_3_ = 6.2 Hz, 2H, NHCH_2_), 3.21 (dd, *J* = 4.3 and 13 Hz, 1H, HCHSH), 2.90 (dd, *J* = 4.3 and 13 Hz, 1H, HCHSH), 2.05 (s, 3H, CH=CHCOCH_3_), 1.97 (s, 3H, CH_3_CO). ^13^C NMR (500 MHz, DMSO): δ 197.05, 173.18, 171.45, 146.23, 142.30, 131.34, 130.09, 128.85, 127.64, 54.15, 46.35, 27.84, 25.72. HRMS: calculated for C_16_H_20_N_2_O_3_S, 320.12; observed [M + Na]^+^, 343.11.

### Nematode Population

Root-knot nematode *M. incognita*(17) and *M. javanica* populations were maintained for 2 months at 25 ± 2 °C on susceptible variety tomato plants (*Solanum lycopersicum* L., cv. Rutgers) in a greenhouse located in Bari, Apulia region, Italy. For the experiments, infested plants were uprooted and roots showing a large number of egg masses and galls were gently washed with tap water to remove soil debris. To collect egg masses, infested roots were cut in small 2 cm pieces and the egg masses were handpicked. The hatching process consisted of placing batches of 20 similar egg masses (averaging 20 000 eggs) on 2 cm diameter sieves (215 μm), which were subsequently put in a 3.5 cm diameter plastic Petri dish. Then, distilled water was added to cover egg masses. The incubating room temperature was kept at 25 °C for the hatching period.^18^ After the first 3 days, hatching second-stage juveniles (J2) were eliminated and only the second-stage juveniles hatched after 24 h or more were collected and used in the *in vitro* nematicidal assays.

### Nematicidal Assay

A total of 17 acetylene derivatives were tested for the nematicidal activity on *M. incognita* second-stage juveniles, and the corresponding EC_50_ values were calculated. Stock solutions of the selected nematicidal compounds were prepared using DMSO, whereas test solutions were obtained by dilution with plain water. To avoid solvent toxicity to nematodes, the final concentration of DMSO in each well never exceeded 1%, a non-toxic concentration for the juveniles. Distilled water with 1% DMSO was used as the control. About 25 *M. incognita* juveniles were used in each replicate treatment in a 96-well plate. Plates were covered with aluminum foil to avoid solvent evaporation and light and kept at 28 °C. After 48 h, juveniles were placed into plain water and sorted out into two groups, motile or immotile, using an inverted microscope at 40× after pricking the body of paralyzed nematodes with a sharp needle. Nematodes that never move after being moved to distilled water and pricked were classified dead. Six replications were made for each concentration, and the experiment was repeated twice. Abamectin and fosthiazate served as positive controls.

### Statistical Analysis

The motility experiments were replicated 6 times, and each experiment was performed twice. The percentages of immotile J2 in the microwell assays were corrected by elimination of the natural death/immotility in the water control according to the formula: corrected % = [(mortality % in treatment – mortality % in control)/(100 – mortality % in control)] × 100. Data were analyzed by analysis of variance (ANOVA) and combined over time. Because ANOVA indicated no significant treatment by time interaction, the means were averaged over all experiments. Corrected percentages of immotile J2 treated with test compounds were subjected to nonlinear regression analysis using the log–logistic equation:^19^*Y* = *C* + (*D* – *C*)/{1 + exp[*b*(log(*x*) – log(EC_50_))]}, where *C* is the lower limit, *D* is the upper limit, *b* is the slope at EC_50_, and EC_50_ is the test compound concentration required for 50% death/immotility of nematodes after elimination of the control (natural death/immotility). In the regression equation, the test compound concentration (%, w/v) was the independent variable (*x*) and the immotile J2 (percentage increase over water control) was the dependent variable (*y*). The mean value of the six replicates per essential oil and compound concentration and immersion period were used to calculate the EC_50_ value.

### Docking Analysis

#### Ligand Preparation

Ligands were docked in the global minimum energy conformation as determined by molecular mechanics conformational analysis. Three-dimensional ligand input structures were generated using Maestro GUI.^20^ The molecules were prepared by the LigPrep tool^21^ and ionized at a pH of 7.4 by Epik. The compounds were used after minimization by OPLS_2005 force field (ff) without further modifications.

#### Protein Preparation

The atomic coordinates of the unbounded protein with Protein Data Bank (PDB) ID 5D80 were imported into Maestro GUI.^20^ The protein was further optimized using the Protein Preparation Wizard at the physiological pH of 7.4,^22^ and missing side chains were added with Prime.^23^

#### Docking and Post-docking

Molecular docking studies were performed using the covalent docking protocol with two precision modes (fast docking mode and thorough docking mode).^24^ The docking grid was defined by centering on CYS284 and occupied a volume of 21.1 × 35.1 × 90.2 (*x*, *y*, *z*) Å. The nucleophilic addition to the triple bond was set as the main reaction. Default settings were applied. Obtained complexes were subjected to a post-docking procedure based on energy minimization and subsequent binding energy calculations. Binding energies were obtained by applying molecular mechanics and continuum solvation models using the molecular mechanics/generalized Born surface area (MM–GBSA) method. The energy was estimated by the OPLS_2005 ff for molecular mechanic energy (MME) and the surface-generalized Born model (SGBM) for polar solvation energy (VSGB) and the apolar solvation factor (GSA).^25^ The resulting complexes were considered for the binding mode graphical analysis with Maestro.^20^

## Results and Discussion

We recently reported on the nematicidal activity of α,β-unsaturated lactones tulipaline A and 5,6-dihydro-2*H*-pyran-2-one^10^ and a series of aromatic aldehydes^8,9^ and ketones,^11^ hypothesizing a strict connection between their biological activity and their ability to affect V-ATPase functionality. In particular, we postulate the involvement of a covalent interaction between the conjugated unsaturation or the carbonyl group of our selected compounds and the nucleophilic thiol groups of the V-ATPase–cysteine residues at the catalytic site of subunit A.^10^ Thus, the formation of not active S-alkylated adducts might be responsible of the V-ATPase impairment.

This initial proof of concept, together with the observation that some alkynoic esters and alkynones induce a chemoselective cysteine modification of unprotected peptides^16,26,27^ also with the reported nematicidal activity of some alkynes on *Pratylenchus coffeae*,^28^ encouraged us to pursue on the investigation of a series of electron-deficient alkynes, with the aim of identifying the structural features required for the explanation of their biological activity. Although the significance of alkyne derivatives in crop protection chemistry is recognized, they are proposed mainly as herbicides, fungicides, insecticides, and acaricides and rarely as nematicides.^29^ Noteworthy, as far as we know, the few examples of natural and synthetic acetylene compounds had been reported for their nematicidal activity without any investigation on the mode of action.^28,30^

A series of differently substituted acetylene derivatives was selected (Figure 1), and for comparison, abamectin and fosthiazate were used as chemical controls.

Many of the tested compounds showed important nematicidal activity (Table 1). Consistent with our previous works on carbonyl compounds, we observed that the treated J2 nematodes were paralyzed or died (filled with liquid in a straight shape).^8−10^

**Table 1:** EC_50_ and Standard Deviation (SD) Values of Individual Compounds against *M. incognita* Calculated at 48 h (*n* = 6) of Immersion in Test Solutions

compound	*M. incognita* (EC_50/48 h_ ± SD, mg/L)
3-butyn-2-one (**1**)	2.38 ± 0.31
4-phenyl-3-butyn-2-one (**2**)	13 ± 0.21
1-chloro-4-phenyl-3-butyn-2-one (**3**)	7.5 ± 1.4
methyl propiolate (**4**)	2.83 ± 0.28
ethyl 2-butynoate (**5**)	>100
ethyl 2-hexynoate (**6**)	75 ± 10
ethyl 2-nonynoate (**7**)	90 ± 16
ethyl 3-phenylpropiolate (**8**)	>100
3-phenylpropiolamide (**9**)	75 ± 12
dimethyl acetylenedicarboxylate (**10**)	1.54 ± 0.16
1-octyne (**11**)	>200
1,7-octadiyne (**12**)	>200
phenylacetylene (**13**)	>200
1,3-diethynylbenzene (**14**)	>200
1-phenylpropyne (**15**)	>200
1-ethynyl-3-methylbenzene (**16**)	>200
1-ethynyl-4-methylbenzene (**17**)	>200
fosthiazate	0.4 ± 0.3
abamectin	0.9 ± 1.6

Although other hypotheses cannot be discarded, this event could represent a possible link to functionally altered V-ATPase, because treated nematodes looked similar to fluid-filled *Caenorhabditis elegans* larvae, in which expression of a V-ATPase gene had been silenced, thus suggesting a critical role of V-ATPase in nematode osmoregulation and detoxification.^31^

Moreover, we postulate that subunit V1 might be principally implicated in the interaction with the presented alkynes, because V_0_-ATPase is specifically involved in the secretion of some components necessary for the cuticle formation of the nematode and that it is independent of proton pumping.^6^

**Scheme 1:**
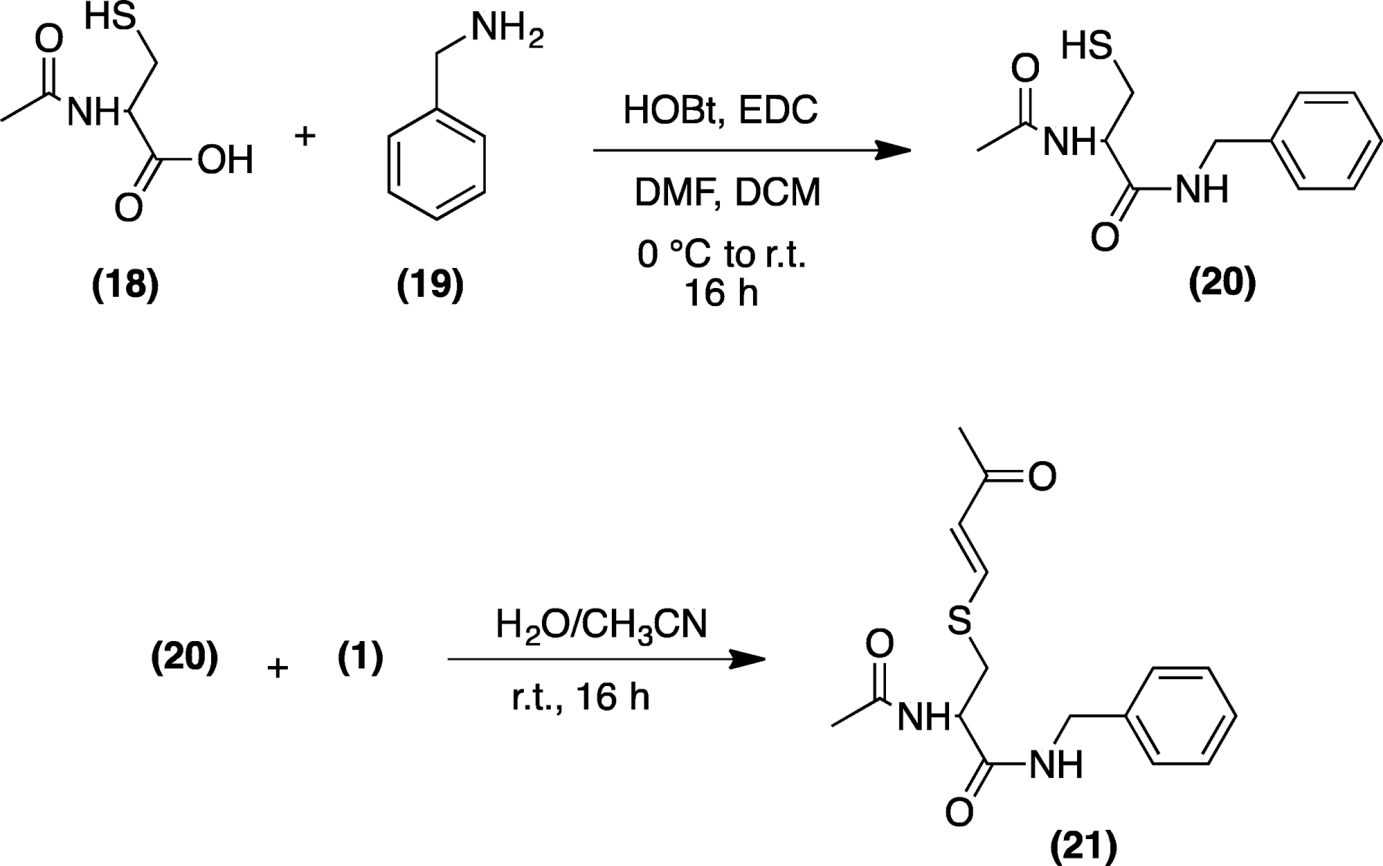
Synthesis of 2-Acetamido-*N*-benzyl-3-[(3-oxobut-1-en-1-yl)thio]propenamide (**21**)

3-Butyn-2-one (**1**) and methyl propiolate (**4**) were two of the best tested compounds, with EC_50/48 h_ of 2.38 ± 0.31 and 2.83 ± 0.28 mg/L, respectively. Interestingly, this similar activity suggests that, if a nucleophilic reaction occurs, the carbonyl group is not involved but, most likely, a 1,4 hetero-Michael addition of protein −SH cysteine residue to the terminal triple bond takes place. To explore this hypothesis, we carried out the reaction between *N*-benzyl-2-acetylamino-3-mercaptopropionamide (**20**) and terminal alkynone (**1**).^16^ The reaction, also followed by means of LC–QTOF–MS, showed only the formation of 2-acetamido-*N*-benzyl-3-[(3-oxobut-1-en-1-yl)thio]propenamide (**21**), therefore demonstrating that the triple bond is the functional group involved (Scheme 1).

Noteworthy, when terminal hydrogen is substituted with an alkyl or aryl group, a dramatic decrease of activity is perceived. The most striking evidence comes from internal alkynoic esters (**5**–**8**) that, with an EC_50/48 h_ of ≥75 mg/L, could be considered relatively less active, especially if compared to methyl propiolate (**4**). A possible explanation might reside in the electron donor behavior of R and Ar substituents, which tackles the activating effect of the ester group conjugated to the triple bond, therefore dramatically affecting the electrophilicity at Cβ.

Similarly, 4-phenyl-3-butyn-2-one (**2**) and 1-chloro-4-phenyl-3-butyn-2-one (**3**) exhibited a lower activity than 3-butyn-2-one (**1**). Interestingly, the introduction of a chlorine atom in the α position to the carbonyl group of compound **2** to generate alkynone (**3**) was shown to be important for activity improvement, as previously noticed in acetophenones,^11^ because one more electrophilic site on C2 is created.

Predictably, two electron-withdrawing substituents at both α positions to the triple bond, as in dimethyl acetylenedicarboxylate (**10**), increased the nematicidal activity, with an EC_50/48 h_ = 1.54 ± 0.16 mg/L.

Besides, to verify our hypothesis, we also selected some acetylene compounds not bearing electron-withdrawing groups. As expected, compounds **11**–**17** were almost inactive (Table 1).

To extend the scope of application of the most active compounds, we decided to accomplish our experiments without the use of organic solvents. Basically, compounds **1**, **4**, and **10** were suspended in water with 0.3% Tween 20 to stabilize the emulsion, and nematicidal activity tests were carried out on both *M. incognita* and *M. javanica*. The stability of the selected compounds in water was also verified by means of ^1^H NMR experiments.

Comparative results are shown in Table 2. Consistent with previous studies,^32,33^ in which analogous differences in paralysis induction susceptibility between those nematode species had been reported, EC_50_ values calculated against *M. incognita* were in all cases lower than the EC_50_ values against *M. javanica*.

**Table 2:** EC_50_ and SD Values of Compounds **1**, **4**, and **10** against *M. incognita* and *M. javanica* Calculated at 48 h (*n* = 6) of Immersion in Test Solutions

	condition	
	H_2_O^a^	H_2_O/DMSO^b^
3-Butyn-2-one (**1**)		
*M. incognita* (EC_50/48 h_ ± SD, mg/L)	3.04 ± 0.33	2.38 ± 0.31
*M. javanica* (EC_50/48 h_ ± SD, mg/L)	5.62 ± 0.95	3.57 ± 0.71
Methyl Propiolate (**4**)		
*M. incognita* (EC_50/48 h_ ± SD, mg/L)	2.50 ± 0.11	2.83 ± 0.28
*M. javanica* (EC_50/48 h_ ± SD, mg/L)	10.22 ± 1.33	5.87 ± 0.70
Dimethyl Acetylenedicarboxylate (**10**)		
*M. incognita* (EC_50/48 h_ ± SD, mg/L)	1.64 ± 0.33	1.54 ± 0.16
*M. javanica* (EC_50/48 h_ ± SD, mg/L)	7.68 ± 1.15	2.64 ± 0.37

a Water with Tween 20 (0.3%).

b DMSO (1%).

Interestingly, alkynone (**1**) and alkynoic esters (**4** and **10**) showed no variation of EC_50_ values against *M. incognita* nematodes in the experiments performed with or without DMSO. This evidence might be related to a possible nematicidal systemic effect rather than a topic alteration of the cuticle structure. Besides, a water formulation is a suitable choice for a potential field application.

Recent studies on covalent small-molecule modulators of the V-ATPase complex identified some ligandable cysteines within the ATP6 V1A component.^34,35^

Specifically, Chen and co-workers reported an interesting electrophilic quinazoline derivative decorated with a carbonyl group and a triple bond that recalls some structural similarities of our acetylene compounds. They also recognized the vacuolar ATPase catalytic subunit A (ATP6 V1A) as the probable target of their “clickable” compound. In particular, by the use of selectively mutated mutants, they identified the cysteine residues most likely involved in the covalent interaction with the ligand.

Thus, to obtain further insight into the probable interaction of our derivatives with those cysteine residues and to gain a deeper comprehension of the key structural aspects of this interaction, we performed docking studies for compounds **1**, **4**, **7**, **8**, and **10**.

We used the crystal structure of yeast V1-ATPase in the autoinhibited form of *Saccharomyces cerevisiae* and performed covalent docking (CovDock) to identify the covalent bonds between each ligand and the receptor.^36^

The procedure comprised a combination of the docking program Glide,^37^ followed by Prime for side-chain rearrangements and minimization of residues within the binding pocket.^38^

The binding box was centered on the CYS284 residue of the A subunit and occupied a volume of 21.1 × 35.1 × 90.2 (*x*, *y*, *z*) Å, which allowed for exploration of the entire ATPase A domain.

**Figure 2:**
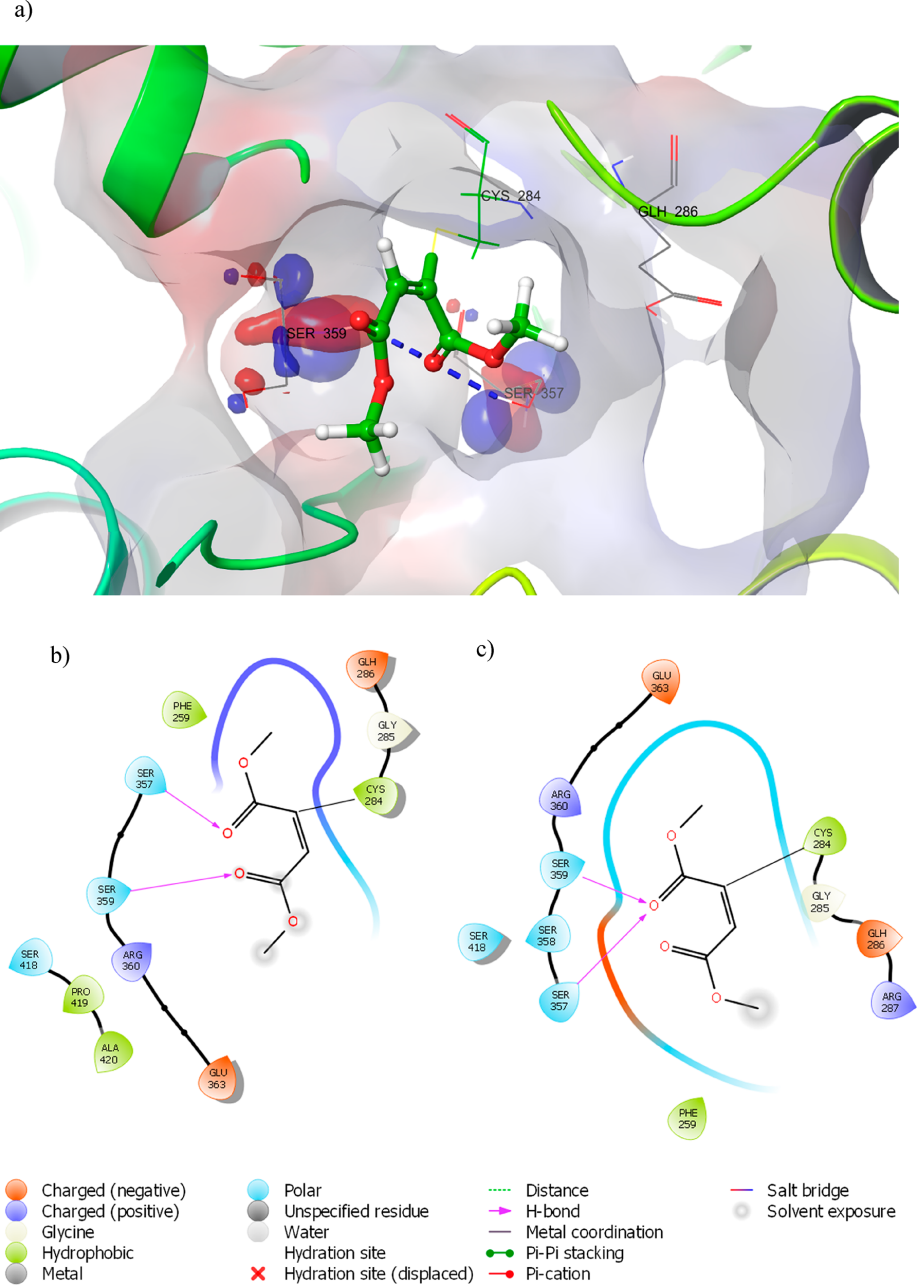
(a) Putative binding mode of compound **10** and two-dimensional (2D) representation of binding-pocket interacting residues for compound **10** in (b) fast docking mode and (c) thorough docking mode.

Docking experiments positioned compounds **1**, **4**, **7**, **8**, and **10**, between β-strand β-21 and α-helix α4, which includes residues 284–287 and 357–360 (Figure 2a). This positioning was mainly due to the covalent bond with CYS284. In detail, the receptor site displayed a small hydrophobic pocket produced by the residues PHE259 and GLY285, GLH286, ARG287 adjacent to CYS284, and a polar portion on the α-helix α7, formed by SER357, SER358, and SER359.

Ethyl 2-noninoate (**7**) and ethyl 3-phenylpropiolate (**8**) showed a low affinity to the receptor site. In particular, compound **8** does not produce any pose, while compound **7** exhibited an unacceptable pose. A possible explanation might be related to the inability of compounds **7** and **8** to accommodate their bulky groups as a result of the steric hindrance effect.

**Figure 3:**
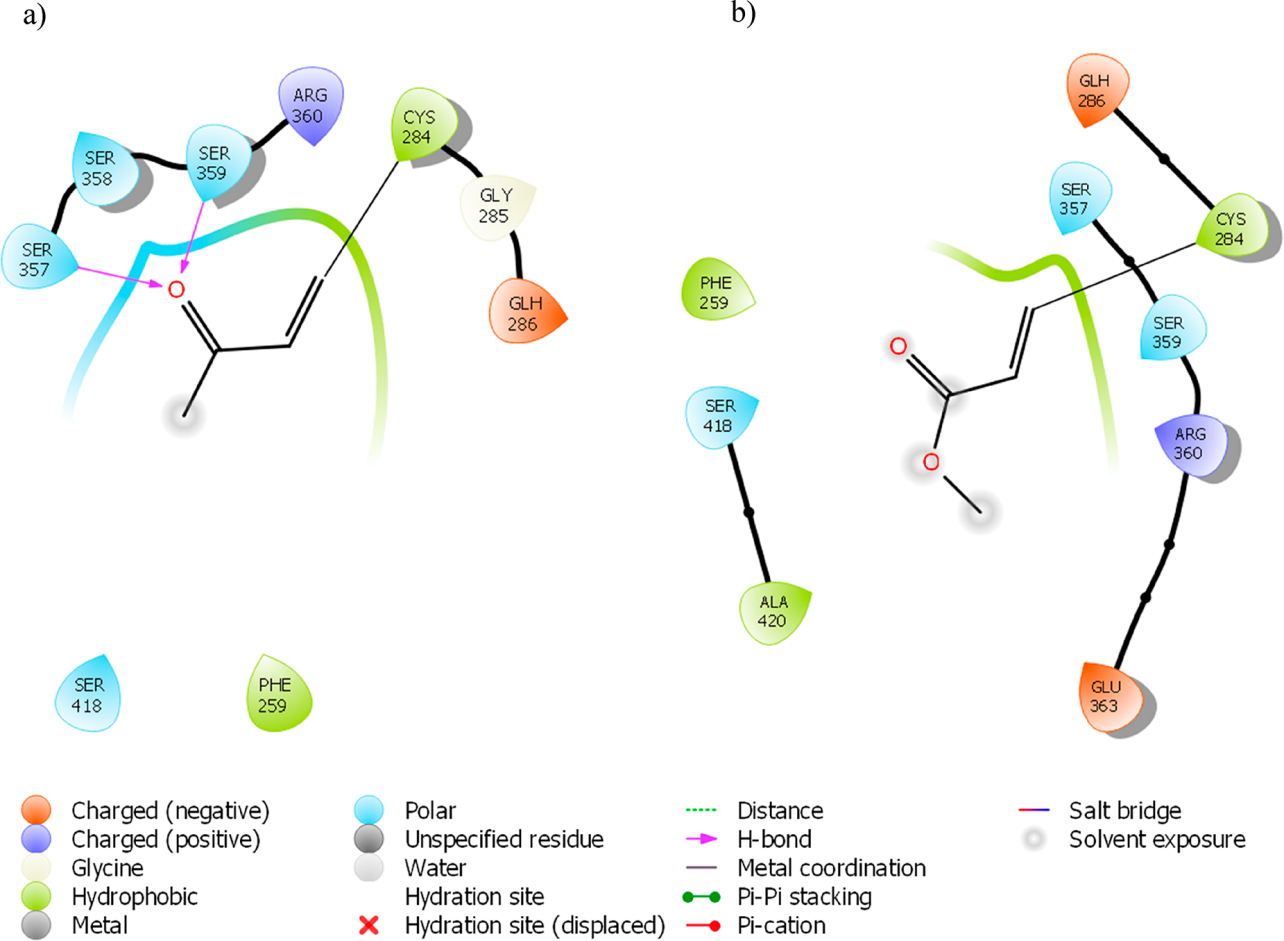
2D representation of binding-pocket interacting residues for compounds (a) **1** and (b) **4** in thorough docking mode.

Dimethylacetylene dicarboxylate (**10**) adopted a slightly different orientation (panels b and c of Figure 2) and, among the selected compounds, was the compound that fit better inside the receptor. Remarkably, the formation of a hydrogen bridge between carbonyl oxygen of the ester group that acts as a hydrogen-bonding acceptor and SER357 and SER359 residues seemed to stabilize the ligand inside the pocket with the best conformational adaptation. The same stabilizing effect was observed for 3-butyn-2-one (**1**) (Figure 3a), while methyl propiolate (**4**) (Figure 3b), although not engaging hydrogen bonds, seemed to fit well in the small pocket as a result of hydrophobic interactions.

The different Glide scores and the variations in the interaction energies justified the differences in the stabilities of these complexes, as shown in Table 3.

**Table 3:** Glide Docking Score and Change in the Total Interaction Energy (Δ*E*_tot_) of [Compound–V-ATPase] Complexes

compound	Glide score (fast docking)	Glide score (thorough docking)	Δ*E*_tot_ (kcal mol^–1^)
**1**	–2.399	–2.513	–29.75
**4**	–2.253	–2.551	–28.18
**7**	0.358	0.151	
**8**			
**10**	–4.084	–3.423	–35.40

All of these proofs of concept demonstrate that acetylene compounds may act as potential pivotal actors in the struggle against the infection of nematodes. Docking studies entailed CYS284 as a key residue for the covalent interaction with the ligand, suggesting that bulky substitution on C3 negatively affects ligand adaptation inside the pocket.

Moreover, the hydrogen-bonding interaction with SER357 and SER359 may usefully stabilize the binding complex. These results are congruent with biological assays that recognized compounds **1**, **4**, and **10** as the most active compounds.

From a chemical point of view, because kinetic data demonstrated that α,β-unsaturated compounds should react preferentially with −SH groups in aminothiols attached to primary carbon atoms,^39^ we postulate that the alkynes presented in the paper probably undergo a Michael reaction, giving rise to S-alkylated adducts.

In conclusion, “electron-deficient” triple-bond moieties may result in a source of key structures for future development of novel synthetic nematicides and the discovery of new molecular target sites.

## Abbreviations Used

DCM: dichloromethane
DMF: *N*,*N*-dimethylformamide
DMSO: dimethyl sulfoxide
HOBt: *N*-hydroxybenzotriazole
EDC: *N*-(3-dimethylaminopropyl)-*N*′-ethylcarbodiimide
LC–QTOF–MS: liquid chromatography quadrupole time-of-flight mass spectrometry
TLC: thin-layer chromatography
THF: tetrahydrofuran
